# The Mysterious Association Between Atrial Fibrillation and Cancer: A Literature Review

**DOI:** 10.7759/cureus.47278

**Published:** 2023-10-18

**Authors:** Mohammed A Miqdad, Lina Alatta, Duaa S Mohamed, Naureen Syed, Mazin Ali, Leina Elomeiri, Amal Alamin, Hina Zubair, Yaseen Abdalla, Nadir Abdelrahman

**Affiliations:** 1 Research, Michigan State University, East Lansing, USA; 2 Nephrology, NewYork-Presbyterian, New York, USA; 3 Nephrology, NewYork-Presbyterian Queens, New York, USA; 4 Internal Medicine, University of Maryland Capital Region, Lake Arbor, USA; 5 Family Medicine/Geriatrics, Michigan State University College of Human Medicine, East Lansing, USA

**Keywords:** atrial fibrillation, cardiac arrhythmia, malignancy risk, colon cancer, cancer

## Abstract

Atrial fibrillation (AF) is a prevalent cardiac dysrhythmia, particularly affecting older adults, with its prevalence rising due to the aging population. AF is linked to several adverse outcomes, including embolic stroke, heart failure, and cancer. The association between AF and cancer is intricate and not yet fully understood. Studies suggest that the rise in cancer survivorship, along with cancer treatments, may contribute to an increased incidence of AF among cancer patients. This literature review was conducted using various databases to explore the relationship between AF and cancer. Studies from 2002 to 2022 were included, focusing on the adult population. Independent authors evaluated and validated the studies, ensuring rigorous methodology. The connection between AF and cancer appears multifaceted. There is evidence of increased cancer incidence within the first few months following an AF diagnosis, with potential shared risk factors like age, obesity, and smoking. Medications used to treat AF, notably amiodarone, were associated with increased cancer risk. Colon cancer risk might be linked to anticoagulation-induced gastrointestinal bleeding. It remains uncertain whether AF diagnosis leads to early cancer detection or if cancer itself contributes to AF development. The complex interplay between AF and cancer involves shared risk factors, potential medication-related influences, and unclear causal directions. The intricacies of this relationship warrant further research to clarify the underlying mechanisms and potential interactions. A comprehensive meta-analysis could provide more insights into this intriguing association and guide future clinical interventions.

## Introduction and background

Atrial fibrillation (AF) is the most common cardiac dysrhythmia in clinical practice, affecting more than 33.5 million people globally, especially older adults. The prevalence of AF has increased three to fourfold over the past 50 years. As the population ages, the prevalence is projected to rise further. Epidemiological data from the Framingham Heart Study indicate a three-to-four-fold increase in AF prevalence over the last 50 years. The adult population older than 65 years is projected to double from 12% in 2010 to 22% by 2040. This increase will likely lead to a rise in the prevalence of AF [1]. The incidence of AF lies between 0.21 and 0.41 per 1000 person-years. Furthermore, 50% of patients with AF have permanent AF, 25% have paroxysmal AF, and 25% have persistent AF [2].

AF is associated with a higher risk of embolic stroke, myocardial infarction, dementia, heart failure, chronic kidney disease, venous thromboembolism, and cancer. Many studies have reported a bidirectional affiliation among myocardial infarction, heart failure, venous thromboembolism, chronic kidney disease, cancer, and incident AF [3]. In recent years, enhanced preventive measures, early detection, and advances in treatment have improved cancer outcomes [4]. However, the risk of developing and dying from cardiovascular diseases has increased among cancer survivors. This is likely because cancer survivors are often older and have other risk factors for cardiovascular disease [5].

Several studies have reported a 20% prevalence of AF in cancer patients, regardless of cancer type [6]. The etiology of AF in cancer patients is likely multifactorial. It has been suggested that the mechanism involved includes the pro-inflammatory immune status, post-treatment inflammatory response to cancer surgery, and cardiotoxic effects of chemotherapy and radiotherapy [7]. The rising incidence of AF among cancer patients is a cause for concern, as it can complicate the clinical course and increase the risk of morbidity and mortality. The economic and emotional burden of AF in cancer patients is also significant, as the lifetime risk of cancer is one in two among people born in the 1960s [6-8].

The increasing use of cancer screening techniques and advanced cancer therapies has led to an increase in cancer survivorship. However, the risk of developing cardiovascular diseases and treatment-related cardiotoxicity has increased among cancer patients. Likewise, multiple reports indicate that cancer patients have a heightened likelihood of AF within the first 90 days following their diagnosis, which increases to fivefold in patients who underwent any surgical procedures [9].

Different cancer subtypes were found to have an association with AF. Some studies showed that multiple myeloma and lung cancer have a strong association with AF specialty in patients younger than 80 years [10]. In older patients aged 80 years or more, it was found that non-Hodgkin's lymphoma and prostate cancer were strongly associated with AF. Other studies showed an increased onset of AF in women with breast cancer, particularly patients with advanced breast cancer stages [11]. Similarly, the prevalence of AF in patients with colorectal cancer is higher than in the normal population, and the prevalence increases with age [12].

## Review

Methods

A literature review of English language papers published between 2002 and 2022 was conducted using PubMed, Google Scholar, ResearchGate, and Web of Science. Clinical trials, case-control studies, cohorts, case series, case reports, systematic reviews, and meta-analysis studies were included. Inclusion criteria included an adult population over 18 years old, articles published in 2002 and onwards, and articles published in English. Exclusion criteria included all articles that did not meet one or more of the inclusion criteria. Five independent authors approved and analyzed the studies to minimize bias, and four reviewed and validated the results. Moreover, any disagreements concerning the eligibility of the publication were discussed among the researchers, and a consensus was met.

The primary endpoint was the risk of colorectal cancer secondary to AF. The secondary endpoint was the risk of all types of cancer secondary to AF. The following keywords were used: "Atrial fibrillation," "Cancer(s)," "Neoplasm(s)," "Malignancy(-ies)," and "Colorectal cancer." This review article aims to identify the possible increase in the risk of colorectal cancer or any cancer in patients with AF and vice versa.

Discussion

Overview and Background

The association between AF and cancer has been reviewed in a systematic review by Lateef et al., which revealed a significant increase in cancer diagnosis within the initial three months after a diagnosis of AF, with only a slight elevation in risk after this period. The potential mechanisms underlying the correlation between AF and an increased risk of long-term cancer remain a mystery. This association may be clarified by sharing the common risk factors, such as advanced age, diabetes, obesity, and smoking, for both cancer and AF. Additionally, antiarrhythmic medications, which are the first-line treatment for AF, have been shown in several studies to increase the risk of cancer, particularly amiodarone [13].

AF and Pharmacological Drugs

Amiodarone use was found to be associated with a higher risk of cancer incidence in male patients (standardized incidence ratio (SIR), 1.18; 95% CI, 1.02 to 1.36; p = 0.022). Similarly, with an odds ratio of 1.60 (1.45-1.77), amiodarone was associated with a higher risk of malignant neoplasm of the liver and intrahepatic bile ducts. Other antiarrhythmic medications were also found to increase the risk of cancer, such as quinidine and propafenone, although the adjusted ratios for these two drugs were not considerable. Moreover, few case reports have proposed the correlation between propafenone use and hepatocellular carcinoma (HCC). The proposed mechanism by propafenone-induced hepatotoxicity via steatohepatitis, hepatocellular injury, and fibrosis leads to cirrhosis and HCC [14].

AF and Cancer Detection

The increased detection of colon cancer may be attributed to gastrointestinal bleeding caused by anticoagulation, as suggested by the prevalence of colon cancer cases. Another study reinforced this by discovering a strong association between a new-onset AF diagnosis and subsequent cancer diagnosis in both males and females in a prospective cohort study. The relative cancer risk was notably elevated for both sexes in the initial 90 days following an AF diagnosis and remained high for the first 365 days [15].

Furthermore, AF was significantly linked to metastatic cancer, particularly within the first 90 days of AF diagnosis. AF was also associated with a higher risk of localized cancer in both sexes throughout the study period, though this was not statistically significant in women. Colorectal and lung cancer risks peaked within the initial 90 days of AF and remained elevated for both sexes over the subsequent 365 days [15].

There could be several explanations for the temporal correlation between an AF diagnosis and a cancer diagnosis. First, AF symptom diagnosis may detect previously hidden cancer. If the cancer remains undetected, AF-related antithrombotic therapy may increase the risk of bleeding, subsequently leading to cancer detection. Evidence has shown that anticoagulant-related gastrointestinal bleeding can reveal cancer within the initial month of treatment, consistent with the increased risk of colorectal cancer diagnosis within the first 90 days after AF diagnosis [15].

Additionally, some treatments used for AF, like amiodarone or digoxin, might be associated with a higher risk of cancer. This raises the question of whether there is an existing connection between AF and cancer. However, considering the notable link to metastatic cancer within the first 90 days, the initial substantial rise in the total number of cancer cases, and the gradual reduction in risk ratios over time, it becomes more probable that cancer may already be present at the time of AF diagnosis [15].

The pathophysiology behind that could be chronic inflammation, which is a major element in the onset and progression of AF, with inflammatory pathways contributing to electrical and structural atrial remodeling [15]. In a large prospective study, it was observed that individuals newly diagnosed with AF had a higher likelihood of subsequently getting diagnosed with cancer [15]. The risk of lung and colorectal cancer was extraordinarily high in the first 90 days after AF diagnosis. Nonetheless, breast or prostate cancer was not associated with AF [15]. Metastatic cancer was more likely to be present during AF diagnosis, and cancer detection may have resulted from increased medical attention [16]. An increased association between AF and localized cancer during the full follow-up period may indicate a common causal pathway [15].

The latest studies discovered a notable inverse association between AF and cancer development in the future. This finding remained consistent in adjusted matched and unmatched analyses and for both colorectal cancer and breast cancer risk. There was no link found between cancer and the development of AF in the future. However, the immediate period (90 days) following a cancer diagnosis was linked to an increased risk of AF [16].

A meta-analysis of 533,514 participants found that patients with a new-onset atrial fibrillation (NOAF) diagnosis have a 24% increased risk of cancer detection in the first 90 days [17]. Moreover, Conen et al. support the earlier studies; even after extensive multivariable adjustment, participants with NOAF had a significantly increased risk of incident cancer during subsequent follow-up in a large prospective cohort study of initially healthy women. The relative increase in risk was greater within three months of NOAF, but smaller increases in risk persisted over time. AF was most strongly correlated to colon cancer among the cancer subtypes studied. Among women with newly diagnosed cancer, the risk of AF increased only during the first three months but not after that [18].

Similarly, Ostenfeld et al.’s cohort study has found that individuals who develop AF for the first time have a notably higher relative risk of being diagnosed with cancer within the initial three months following their AF diagnosis. The team found strong links between AF and lung, kidney, and colon cancers. Furthermore, AF was also strongly linked to metastatic cancer. Nonetheless, the absolute cancer risk within the first three months was only 2.5%. After three months of follow-up, there was a little increase in overall cancer risk (SIR, 1.13; 95% CI, 1.12-1.15) [19].

The sharp decrease in relative risks after the initial three months of follow-up implies that the cancers likely existed at the time of the AF diagnosis rather than resulting from AF. It is possible that these findings were influenced by increased medical surveillance among patients with newly diagnosed AF. A thorough clinical examination and screening for underlying diseases are part of the diagnostic work-up for AF or conditions caused by AF, which may lead to cancer detection. For instance, in individuals suspected of having a stroke due to AF, a chest X-ray might detect early-stage lung cancer, while a brain scan may detect early-stage brain cancer [19]. The Oulu Project Elucidating Risk of Atherosclerosis (OPERA), a study of over 1000 participants with a longer follow-up period of 16.3 years, found that cancer development was an independent risk factor for AF. It is still unclear whether this link is causal or if cancer and AF share the same pathophysiologic mechanisms [19].

Cancer and AF share common risk factors such as age, gender, and smoking. Despite controlling these factors, the link between AF and cancer remains, and additional mechanisms are required. Previous research has linked cancer surgery, medical therapy, inflammation, autonomic nervous system imbalance, paraneoplastic manifestations, and other cancer-related comorbidities to an increased risk of AF in cancer patients [20]. Despite the multifaceted research, the association between AF and cancer appears very complex and needs further research as many common risk factors, biology, and common pathways such as systemic inflammation, pain-mediated autonomic system dysfunction, oxidative stress, mechanical stress, and local inflammation may potentially predispose to both tumorigenesis and AF development. Also, some grey areas need further research, especially the frequency of ischemia, bleeding complications, and management of antithrombotic therapy according to cancer site and stage in AF patients [21].

## Conclusions

Cancer and AF share the same risk factors: age, gender, alcohol consumption, and smoking. There could be a mysterious association between cancer and AF; however, the association remains ambiguous. Does AF diagnosis lead to early detection of cancer, or does cancer itself lead to AF, remain unresolved questions. A meta-analysis to answer such questions is highly required.
